# Effects of the COVID-19 Pandemic on Humeral Shaft Fracture Management and Its Outcomes

**DOI:** 10.7759/cureus.43433

**Published:** 2023-08-13

**Authors:** Chrystina L James, Trevor D Wolterink, Bushra Fathima, Gabriel B Burdick, Susan G Wager, Jager W Haan, Yash D Hegde, Stephanie Muh

**Affiliations:** 1 Department of Orthopaedic Surgery, Henry Ford Health System, Detroit, USA; 2 Department of Orthopaedic Surgery, Wayne State University School of Medicine, Detroit, USA; 3 Department of Orthopaedic Surgery, Michigan State University College of Human Medicine, East Lansing, USA; 4 Department of Orthopaedic Surgery, Henry Ford Health System, West Bloomfield, USA

**Keywords:** pandemic, covid-19, humeral shaft fracture, fracture, humerus

## Abstract

Background and objective

The coronavirus disease 2019 (COVID-19) pandemic necessitated a sudden and drastic shift in patient management throughout the healthcare system, to curb the spread of the disease and deal with resource limitations. Many surgical cases were canceled or delayed with only the most urgent and emergent cases taken up for treatment. It is unknown if and how these alterations affected patient outcomes. The purpose of this study was to compare time to fracture care and outcomes between patients treated for humeral shaft fractures prior to the COVID-19 pandemic and those treated during the pandemic. We hypothesized that the pandemic cohort would have a prolonged time to fracture care and worse outcomes than the pre-pandemic cohort.

Materials and methods

This was a retrospective cohort study performed within a single healthcare system. All humeral shaft fractures treated from March to June 2019 (pre-pandemic cohort) and March to June 2020 (pandemic cohort) were identified using International Classification of Diseases, Ninth Revision, Clinical Modification (ICD-9-CM) codes and ICD-10-CM codes as well as Current Procedural Terminology (CPT) codes. Data on demographics, fracture characteristics, treatment, and outcomes were collected via chart and radiograph review. Outcomes analyzed included time to being made weight-bearing as tolerated (WBAT), radiographic union, and final follow-up; range of motion (ROM) at radiographic union and final follow-up; and rate of complications.

Results

The pre-pandemic cohort (n=19) was significantly younger with a mean age of 29 years than the pandemic cohort (n=17) with a mean age of 49 years (p=0.010). There were no other significant differences in demographics, fracture characteristics, or treatment type between the groups. Time to fracture care was not significantly different in the pre-pandemic cohort (five days) versus the pandemic cohort (four days). Time to being made WBAT, radiographic union, and final follow-up were not significantly different between the pre-pandemic (86, 113, and 98 days) and the pandemic cohorts (77, 106, and 89.5 days). ROM measurements in abduction at radiographic union were significantly different between the cohorts: in the pre-pandemic cohort, 100% of patients reached greater than 160 degrees; in the pandemic cohort, only 16.7% of patients reached greater than 160 degrees (p=0.048). There was a non-significant decrease in the proportion of patients who achieved the maximal category of ROM measurements in forward elevation and extension at radiographic union and abduction, forward elevation, and extension at final follow-up, as well as a non-significant increase in visual analog scale (VAS) pain scores at final follow-up between cohorts. There were no significant differences in the rate of complications.

Conclusions

Despite limited resources, reduced operating room availability, and increased utilization of virtual visits due to the COVID-19 pandemic, patients with humeral shaft fractures may not have faced delays in fracture care or worse outcomes compared to the pre-pandemic period. The pandemic cohort may have experienced significantly decreased ROM compared to the pre-pandemic cohort, which may reflect the decreased availability of physical therapy services and overall decreased activity levels due to the quarantine orders. However, we could not identify any other significant differences in the type of treatment, pain, complications, or time to union.

## Introduction

After a few months into the global spread of severe acute respiratory syndrome coronavirus 2 (SARS-CoV-2), the World Health Organization (WHO) declared coronavirus disease 2019 (COVID-19) a pandemic on March 11, 2020, leading to drastic changes in healthcare systems worldwide [1]. To respond to the rapidly increasing number of patients hospitalized with COVID-19 and quickly dwindling supplies, many healthcare systems were forced to reorganize their operations to curb the spread of the virus and preserve limited resources.

One facet of this response involved the postponement of non-emergent surgeries across most if not all surgical specialties in the United States, resulting in a large decrease in the overall surgical volume [2-4]. However, due to the urgent or emergent nature of many traumatic orthopedic injuries, some level of orthopedic care had to be continued even as most elective procedures were delayed or canceled [5-10]. The early months of the pandemic also witnessed an exponential increase in the use of virtual care and the utilization of telehealth, enabling a large portion of outpatient care to be performed remotely [11]. Despite the concerted efforts to continue performing essential surgeries and ensure appropriate follow-up, COVID-19 disrupted healthcare and society in innumerable ways, and the outcomes of acute care that was delivered during the pandemic may have been broadly impacted.

The purpose of this study was to determine the effects of the pandemic on the management and outcomes of a common orthopedic injury, humeral shaft fractures. Humeral shaft fractures represent 2-4% of all fractures and have historically been managed nonoperatively with bracing, splinting, or casting; however, operative management of this condition has been on the rise over the last several decades [12,13]. The existing literature does not indicate any clear superiority of one treatment over another. Numerous studies have shown that nonoperative treatment leads to higher rates of malunion and nonunion, but no differences in time to clinical union (absence of fracture site pain and motion), final range of motion (ROM), functional outcomes, and patient satisfaction have been documented [12-14]. In both operative and nonoperative treatment, studies have shown that time to fracture care, whether by surgery or brace application, is very important for the progression of fracture union [12,14].

The primary outcome measured in this study was time to fracture care following a humeral shaft fracture. Secondary outcomes included time to radiographic union, time to being made weight-bearing as tolerated (WBAT), ROM at the time of radiographic union and at final follow-up, and rate of complications. We hypothesized that patients who underwent treatment for diaphyseal humerus fractures during the initial surge of the COVID-19 pandemic (March-June 2020) experienced delayed care and poorer outcomes compared to a 2019 pre-pandemic cohort.

## Materials and methods

This study was conducted at a large health system consisting of five hospitals in both urban and suburban settings. All humeral shaft fractures treated between March and June of 2019 (classified as the pre-pandemic cohort) and March and June of 2020 (pandemic cohort) were identified by querying the electronic medical records for International Classification of Diseases, Ninth Revision, Clinical Modification (ICD-9-CM) codes and ICD-10-CM codes as well as Current Procedural Terminology (CPT) codes. The inclusion criteria were ICD-9 code 812 (fracture of humerus), ICD-10 code S42.3 (fracture of shaft of humerus), or CPT codes 24500, 24505, 24515, or 24516 (closed treatment of humeral shaft fracture without manipulation, closed treatment of humeral shaft fracture with manipulation, open treatment of humeral shaft fracture with plates/screws, or treatment of humeral shaft fracture with insertion of intramedullary implant, respectively). This study was approved by our institutional review board.

Patients were excluded if they were under the age of 18 at the time of injury, had bilateral humeral shaft fractures, pathologic fractures, periprosthetic fractures, intra-articular fractures, or a history of a previous ipsilateral humeral shaft fracture, following which 19 patients were included in the pre-pandemic cohort and 17 patients in the pandemic cohort.

A retrospective chart review was performed to collect demographic information including age, gender, race, marital status, employment status, insurance type, BMI, and smoking status. Provider notes and radiographs were reviewed to assess fracture characteristics such as laterality (dominant vs. non-dominant side), location (proximal-, mid-, or distal-third diaphysis), associated polytrauma, and if the fracture was open or closed. Injury mechanism, date of injury, date of fracture care, type of treatment, and type of follow-up visit (virtual or in-person) were also recorded. Treatment was either nonoperative or operative, consisting of open reduction and internal fixation (ORIF) or intramedullary nailing (IMN). The date of fracture care was considered to be the first outpatient clinic appointment for patients treated nonoperatively and the date of surgery for patients treated operatively. Radiographic union, defined as bridging callus on three cortices, was determined by assessing radiographs taken at routine follow-up appointments; all radiographs were analyzed by the same member of the study team. ROM measurements (shoulder forward elevation and abduction; elbow flexion and extension) were recorded at the radiographic union as well as at the final follow-up. Shoulder ROM was classified as achieving full forward elevation or abduction (greater than or equal to 160 degrees for the purpose of this study) and elbow ROM was classified as full flexion (greater than or equal to 120 degrees), full extension (less than or equal to 5 degrees), and full functional arc of motion (greater than or equal to 100 degrees). Complications of treatment, conversion to operative treatment in patients initially treated nonoperatively, the date the patient was made WBAT, and visual analog scale (VAS) pain scores (measured on a scale of 0-10) were also recorded.

Statistical analysis

Descriptive statistics were calculated with categorical data reported as frequencies with percentages and continuous data as medians. Categorical data was analyzed using Fisher’s exact or Fisher-Freeman-Halton test, and continuous data was analyzed using the Mann-Whitney U test. A p-value <0.05 was considered statistically significant. All statistical analyses were performed using IBM SPSS Statistics 28.0.1.1 (IBM Corp., Armonk, NY). Post hoc power analysis was performed using G*Power 3.1 (UCLA, Los Angeles, CA).

## Results

The pre-pandemic cohort consisted of 19 patients (nine males and 10 females) while the pandemic cohort consisted of 17 patients (four males and 13 females). The pre-pandemic cohort with a median age of 29 years was significantly younger than the pandemic cohort with a median age of 49 years (p=0.010). The cohorts were not significantly different in terms of gender, race, marital status, BMI, smoking status, insurance type, or employment status (Table 1).

**Table 1:** Demographics of pre-pandemic and pandemic cohorts BMI: body mass index; Q1: first quartile; Q3: third quartile

Variable		Pre-pandemic cohort (19 patients)	Pandemic cohort (17 patients)	P-value
Age, years	Median (Q1, Q3)	29.0 (21.5, 43.5)	49.0 (36.0, 64.0)	0.01
Gender	Female	10 (52.6%)	13 (76.5%)	0.177
	Male	9 (47.4%)	4 (23.5%)	
Race	Caucasian	10 (52.6%)	13 (76.5%)	0.307
	African American	7 (36.8%)	4 (23.5%)	
	Other	2 (10.6%)	0 (0%)	
Marital status	Single	15 (78.9%)	7 (41.2%)	0.083
	Married	3 (15.8%)	7 (41.2%)	
	Other	1 (5.3%)	3 (17.7%%)	
BMI, kg/m^2^	Median (Q1, Q3)	24.4 (22.3, 31.8)	26.0 (24.7, 36.8)	0.257
Smoking status	No	8 (42.1%)	12 (70.6%)	0.134
	Yes	10 (52.6%)	5 (29.4%)	
	Unknown	1 (5.3%)	0 (0%)	
Insurance type	Medicaid/Medicare	9 (47.4%)	6 (35.3%)	0.458
	Private	7 (36.8%)	10 (58.8%)	
	Other	3 (15.8)	1 (5.9%)	
Currently employed	Yes	12 (63.2%)	8 (47.1%)	0.503
	No	7 (36.8%)	9 (52.9%)	

There were no significant differences in fracture characteristics between the groups, as shown in Table 2. The most common injury mechanisms in both cohorts were ground-level falls and motor vehicle accidents. Time to fracture care, which was determined as the number of days between the date of injury and the date of fracture care, was not significantly different between cohorts: five days in the pre-pandemic cohort vs. four days in the pandemic cohort. The most common fracture location was mid-diaphysis and the majority of fractures were closed in both cohorts. The cohorts were not significantly different with regard to the presence of a polytraumatic injury.

**Table 2:** Fracture and treatment characteristics of pre-pandemic and pandemic cohorts MVA: motor vehicle accident; ORIF: open reduction and internal fixation; Q1: first quartile; Q3: third quartile

Variable		Pre-pandemic cohort (19 patients)	Pandemic cohort (17 patients)	P-value
Time to fracture care, days	Median (Q1, Q3)	5.0 (1.5, 10.5)	4.0 (2.0, 10.0)	0.975
Mechanism	Fall	7 (36.8%)	10 (58.8%)	0.333
	MVA	9 (47.4%)	4 (23.5%)	
	Other	3 (15.8%)	3 (17.7%)	
Location, diaphyseal third	Proximal	2 (10.5%)	3 (17.6%)	0.897
	Mid	12 (63.2%)	9 (52.9%)	
	Distal	5 (26.3%)	5 (29.4%)	
Polytrauma	No	14 (73.7%)	12 (70.6%)	1
	Yes	5 (26.3%)	5 (29.4%)	
Dominant hand	No	13 (68.4%)	8 (47.1%)	0.975
	Yes	4 (21.1%)	9 (52.9%)	
	Unknown	2 (10.5%)	0 (0%)	
Open or closed	Closed	17 (89.5%)	14 (82.4%)	0.65
	Open	2 (10.5%)	3 (17.6%)	
Treatment	Operative	11 (57.9%)	12 (70.6%)	0.502
	Nonoperative	8 (42.1%)	5 (29.4%)	
Type of operative treatment	ORIF	10 (90.9%)	12 (100%)	0.47

Treatment was not significantly different between cohorts with operative treatment being the most common in the pre-pandemic (11/19 patients) and pandemic (12/17 patients) cohorts. The type of operative treatment was also not significantly different with all operative patients in the pandemic cohort undergoing ORIF and 10/11 operative patients in the pre-pandemic cohort also undergoing the same; one patient in this cohort was treated with IMN (Table 2).

As demonstrated in Table 3, there was no significant difference in time to WBAT, with a median of 86.0 days in the pre-pandemic cohort and 77.0 days in the pandemic cohort. The cohorts did not differ significantly in days to the radiographic union, which was 113.0 days in the pre-pandemic cohort and 106.0 days in the pandemic cohort. Length of follow-up, determined as the number of days from the date of fracture care to the date of final follow-up, was 98.0 days in the pre-pandemic cohort and 89.5 days in the pandemic cohort, which was not significantly different. There was also not a significant difference in the type of follow-up appointments (virtual or in-person) between cohorts. In the pre-pandemic cohort, no patient had a virtual visit compared to three patients with at least one virtual visit in the pandemic cohort (p=0.095).

**Table 3:** Outcomes in pre-pandemic and pandemic cohorts ORIF: open reduction and internal fixation; Q1: first quartile; Q3: third quartile; VAS: visual analog scale

Variable		Pre-pandemic cohort (19 patients)	Pandemic cohort (17 patients)	P-value
Time to weight bearing as tolerated, days	Median (Q1, Q3)	86.0 (48.0, 133.0)	77.0 (27.5, 84.5)	0.098
Time to the radiographic union, days	Median (Q1, Q3)	113.0 (90.0, 154.0)	106.0 (87.0, 142.0)	0.605
Length of follow-up, days	Median (Q1, Q3)	98.0 (46.0, 223.0)	89.5 (43.8, 256.3)	0.763
VAS at final follow-up	Median (Q1, Q3)	0 (0, 5.0)	2.0 (0, 5.0)	0.382
Complications	No	18 (94.7%)	13 (76.5%)	0.167
	Yes	1 (5.3%)	4 (23.5%)	
Conversion to ORIF from nonoperative treatment	No	7 (87.5%)	3 (60.0%)	0.51
	Yes	1 (12.5%)	2 (40.0%)	

Regarding ROM, it is important to note that the data was not available for all patients. At the time of radiographic union, only shoulder abduction was significantly different between cohorts (p=0.048). In the pre-pandemic cohort, four patients had this information available and all four patients achieved full shoulder abduction at radiographic union compared to one of six patients in the pandemic cohort who had this information available. There were no other significant differences in ROM at the time of radiographic union (Table 4). The proportion of patients achieving full motion at the time of final follow-up decreased between the pre-pandemic and pandemic cohorts in shoulder forward elevation, shoulder abduction, elbow extension, and elbow functional arc of motion, but none of these decreases were statistically significant (Table 5). 

**Table 4:** Range of motion at the time of radiographic union ROM: range of motion

Variable	Response	Pre-pandemic cohort (19 patients)	Pandemic cohort (17 patients)	P-value
Full shoulder forward elevation (>160 degrees) at radiographic union	Yes	4 (50.0%)	1 (16.7%)	0.301
	No	4 (50.0%)	5 (83.3%)	
Full shoulder abduction (>160 degrees) at radiographic union	Yes	4 (100%)	1 (16.7%)	0.048
	No	0 (0%)	5 (83.3%)	
Full elbow flexion (>120 degrees) at radiographic union	Yes	6 (75.0%)	3 (75.0%)	1.000
	No	2 (25.0%)	1 (25.0%)	
Full elbow extension (0-5 degrees) at radiographic union	Yes	8 (100%)	2 (50.0%)	0.091
	No	0 (0%)	2 (50.0%)	
Elbow functional ROM (>100 degree arc of motion) at radiographic union	Yes	7 (87.5%)	4 (100%)	1.000
	No	1 (12.5%)	0 (0%)	

**Table 5:** Range of motion at the time of final follow-up ROM: range of motion

Variable	Response	Pre-pandemic cohort (19 patients)	Pandemic cohort (17 patients)	P-value
Full shoulder forward elevation (>160 degrees) at final follow-up	Yes	8 (57.1%)	3 (30.0%)	0.240
	No	6 (42.9%)	7 (70.0%)	
Full shoulder abduction (>160 degrees) at final follow-up	Yes	7 (87.5%)	3 (33.3%)	0.050
	No	1 (12.5%)	6 (66.7%)	
Full elbow flexion (>120 degrees) at final follow-up	Yes	9 (75.0%)	6 (75.0%)	1.000
	No	3 (25.0%)	2 (25.0%)	
Full elbow extension (0-5 degrees) at final follow-up	Yes	12 (100%)	6 (75.0%)	0.147
	No	0 (0%)	2 (25.0%)	
Elbow functional ROM (>100 degree arc of motion) at final follow-up	Yes	11 (91.7%)	6 (75.0%)	0.537
	No	1 (8.3%)	2 (25.0%)	

There were no significant differences between cohorts in the remaining outcome measures, including VAS pain score at the last follow, rate of complications, and conversion to operative treatment in patients initially treated nonoperatively (Table 3).

Results of a post hoc power analysis indicated that Fisher's exact or Fisher-Freeman-Halton test utilized for full shoulder forward elevation at radiographic union and final follow-up, full elbow flexion at radiographic union and final follow-up, full elbow extension at final follow-up, elbow functional ROM at radiographic union and final follow-up, and complications was underpowered (3.2-64.6%) for detecting a medium effect at a significance criterion of α=0.05. Similarly, the Mann-Whitney U test utilized for the length of follow-up, time to WBAT, time to radiographic union, and VAS at final follow-up was underpowered (27.0%) for detecting a medium effect at a significance criterion of α=0.05. Tests for all other outcome measures were adequately powered.

## Discussion

The most important finding of this study was that despite the significant changes implemented in the healthcare system in the early months of the COVID-19 pandemic, patients with humeral shaft fractures may have been treated efficiently and effectively without any delays in care or major differences in outcomes when compared to their pre-pandemic counterparts. This finding suggests that we can accept our null hypothesis.

With regard to our primary outcome of time to fracture care, we did not find a significant difference between patients treated during the first months of the COVID-19 pandemic and patients treated prior to the onset of the pandemic. Our institution did not have a specific protocol change in the treatment of traumatic injuries, but as at many hospitals, elective surgeries were canceled in our healthcare system from March to June 2020; however, urgent and emergent cases were prioritized and allowed to proceed. Despite the greatly reduced clinical capacity, resource limitations, and logistical challenges, our study found no delays in care for patients with humeral shaft fractures. Additionally, despite many of our outpatient clinics adding the option of telehealth, there was not a significant increase in the utilization of virtual visits in the follow-up period of this study.

Broadly, telehealth saw an exponential increase in utilization in the early months of the COVID-19 pandemic; one large study found that telehealth use peaked in April 2020 with up to 58% of surgeons using it in some context. However, this same study showed that only 26.8% of surgeons used it for new patient visits [11]. There is also research suggesting that although patients were largely satisfied with using telehealth specifically for musculoskeletal complaints, most would prefer in-person visits [15]. Disparities in care and access exist in telemedicine as in traditional healthcare with data suggesting significantly decreased telehealth visits among patients who are non-Caucasian, male, and have non-commercial health insurance plans [16]. Thus, the non-significant use of virtual visits seen in the present study is likely multifactorial in nature and may be related to the nature of traumatic injuries in addition to the demographics of our patient population.

The authors are unaware of any existing studies examining the management of humeral shaft fractures specifically during the COVID-19 pandemic; however, there are several studies examining overall orthopedic trauma volume as well as rates of surgical intervention. A multicenter retrospective study of hospitals in the United Kingdom found a 34% reduction in referrals for orthopedic trauma and a 29.5% decrease in orthopedic surgical interventions overall [17]. Another study in the United Kingdom found a 43.2% reduction in orthopedic trauma surgery from 2019 to 2020 due to the pandemic [18]. In France, the average age of orthopedic trauma patients increased during the pandemic, but the rate of operative intervention significantly decreased [6]. In contrast, a study at a regional Australian hospital found a significantly higher number of orthopedic referrals during 2020 compared to 2019, but a significantly lower number of operative treatments [19]. Our study found no differences in the type of treatment patients received between cohorts with operative management being more common than nonoperative management in both cohorts.

Since the onset of the pandemic, there have been ongoing discussions regarding the risks of delayed care and higher complication rates that may subsequently be seen. The majority of the data that currently exists to support this examines outcomes in patients who were found to be COVID-19-positive at the time of their injury or surgery. In these patients, several studies support higher mortality and complication rates [7-9]. However, there is very little literature examining complications or delays in non-elective care in COVID-19-negative patients. This study found that patients with humeral shaft fractures experienced similar outcomes overall before and during the pandemic; there were no significant differences between cohorts in any of the outcome measures analyzed. Particularly, the time from fracture care to radiographic union and to being made WBAT were not significantly different in the pandemic cohort compared to the pre-pandemic cohort.

This study also examined ROM at the time of radiographic union as well as at the last follow-up appointment. There were no significant differences in shoulder forward elevation, elbow flexion, elbow extension, or achieving a functional arc of motion of the elbow at either time point. However, the rate of achieving full shoulder abduction was significantly lower in the pandemic cohort than in the pre-pandemic cohort at both time points. This may be a result of both decreased physical activity during the pandemic as well as decreased access to in-person physical therapy due to the mandatory lockdown [20,21]. According to the American Physical Therapy Association, there was an increase in telehealth physical therapy visits during the pandemic, and the clinics that did stay open for in-person visits saw a much lower volume of patients [21].

There are several limitations to this study. Primarily, this was a retrospective study, and thus there was variability in follow-up length and intervals, surgeon experience, operative technique, and documentation, as well as in treatment, postoperative, and rehabilitation protocols. While there were no significant differences in the length of follow-up between cohorts, some of the follow-up care in the pandemic cohort was performed virtually and may not have been able to collect the same information as at an in-person visit. In addition, radiographic union was assessed on radiographs taken at routine follow-up appointments and may not be reflective of the exact length of time until radiographic union. Another major limitation of this study was the small patient population in each cohort. Post hoc power analysis found that the study was underpowered for multiple outcome measures; however, this population size was comparable to that in other similar studies performed in the early months of the COVID-19 pandemic. Lastly, this study was conducted in a single healthcare system, and hence the patient population may not be representative of the general population. However, our study population did include both urban and suburban populations with demographics that are consistent with those that have been previously reported [22-24].

## Conclusions

Our findings revealed that despite the challenges imposed by the COVID-19 pandemic, patients treated for humeral shaft fractures may not have experienced delays in care, prolonged time to radiographic union, or prolonged time to weight bearing when compared to those treated before the pandemic. Patients also may have achieved similar ROM in both cohorts with the exception of shoulder abduction; a lower proportion of patients in the pandemic cohort achieved full abduction. This may be reflective of decreased activity and decreased availability of physical therapy due to the mandatory lockdowns. Overall, patients treated during the early months of the COVID-19 pandemic may have received similar care with similar outcomes and no increase in the rate of complications.
